# Effect of adiponectin gene polymorphisms on waist circumference in patients with diabetes

**DOI:** 10.1186/2251-6581-11-14

**Published:** 2012-09-14

**Authors:** Shirin Hasani-Ranjbar, Mahsa M Amoli, Ozra Tabatabaei-Malazy, Yalda Rumi, Javad Tavakkoly-Bazzaz, Hilda Samimi, Elnaz Abbasifarid

**Affiliations:** 1 Endocrinology and Metabolism Research Center, Tehran University of Medical Sciences, Tehran, Iran; 2 Faculty of Medicine, Tehran University of Medical Sciences, Tehran, Iran; 3 Endocrinology and Metabolism Research Institute, 5th floor, Dr. Shariati Hospital, North Kargar Ave., Tehran 14114, Iran

**Keywords:** Adiponectin, Polymorphism, Type 2 diabetes, Abdominal obesity

## Abstract

**Background:**

Plasma levels of adiponectin which is secreted from adipose tissue are associated with various parameters of metabolic syndrome. This effect seems to be a result of interactions between genetic and environmental factors including central obesity. The present study was carried out to investigate the possibility of relation between single nucleotide polymorphisms of adiponectin gene (+45 T/G and −11391 G/A) and waist circumferences (WC) in patients with type 2 diabetes.

**Methods:**

This cross-sectional study was conducted on n = 238 diabetic patients selected as cases and n = 159 as healthy control who were recruited from Rafsanjan city in south – east of Iran. The possible association of +45 T/G and –11391 G/A adiponectin gene polymorphisms with WC according to age and sex was evaluated.

**Results:**

There was no significant difference in distribution of frequencies of +45 T/G and –11391 G/A adiponectin gene polymorphisms in each group. We only found a significant association between –11391 G/A adiponectin gene polymorphism with WC in diabetic group (p = 0.021). This association was remained significant after adjustment in multivariate regression model (p = 0.019, OR: 0.244, 95%CI: 0.075-0.791) and also this effect was independent of sex and age.

**Conclusion:**

We found higher abdominal obesity in GA or AA carriers of adiponectin – 11391 G/A genotype in type 2 diabetes patients independent of age and sex.

## Introduction

Obesity and diet-dependent development of metabolic syndrome are considered as a global health problem [1,2]. Adiponectin is an adipokyne which is secreted from exclusive differentiated adipose tissue and its plasma level is reduced with increasing of body fat mass [3,4]. It has metabolic effects on fat and glucose metabolism [5] besides anti inflammatory [3], and anti atherogenic roles [6]. Adiponectin exhort its effects through the sensitization of body to the insulin [7,8]. According to the several studies, the plasma level of adiponectin could be reduced in the animal models of obesity [9,10] and also in human obesity particularly in visceral obesity [11-13]. Reduction of adiponectin in plasma is dependent on several factors such as reducing its production or increasing its blood clearance by liver. In addition to genetic factors which are influencing plasma level of adiponectin, some environmental factors such as consumption of the high-fat diet delays the clearance of adiponectin from blood [14] while a rich carbohydrates diet is associated with low levels of adiponectin [15]. In a number of previous studies the relationship between adiponectin genes single nucleotide polymorphisms (SNPѕ) with serum adiponectin level have been reviewed [16-18]. It is generally assumed that type 2 diabetes is caused by both genetic disorder (SNPs) and environmental factors including central obesity [19-21]. In previous reports the association between adiponectin gene polymorphisms with incidence of type2 diabetes in lean subjects was non-significant [22] , but the association between adiponectin polymorphisms and type 2 diabetes and obesity was different [18,19,23-26]. Several polymorphisms within Adiponectin gene have been identified including polymorphisms at position −11391 G/A, -11377 G/C, +45 T/G and 276 G/T [27] The goal of this study was to examine the relation between two polymorphisms in adiponectin gene (+45 T/G and -11391 G/A) and type 2 diabetes in an Iranian population.

## Methods

### Study population

This was a case–control study. The case group included 238 patients with type 2 diabetes who were selected randomly among patients referred to Rafsanjan diabetes clinic. Control group were 159 healthy people who have been chosen from the same population. (The sample of the population is the same as previous studies from our group) [23,28]. Diabetes diagnosis laid according to criteria of America Diabetes association [29].

### Data collection

After obtaining written consent from patients, a questionnaire comprising personal and demographic information for each participant was completed. Waist measurement was performed by a constant co-worker by measuring the widest area which is located between lower edge of lower rib and iliac crest and the unit was centimeter. Abdominal obesity was considered as a wais circumference over 90 cm. Waist circumference has been considered as an index for abdominal obesity. In this study, according to results of other studies which were performed on Iranian population, measures more than 90 cm were considered as abdominal obesity [20,21,30-33]. 3-5 cc blood sample was taken from each subject in tubes containing EDTA and Blood was stored in 20°c. This study was approved by the research ethics committee of Tehran University of Medical Sciences.

### Genotyping

DNA extraction and molecular analysis of adiponectin gene polymorphism: DNA was extracted from blood samples which were collected in tubes containing EDTA through salting out method. Molecular analysis of the +45 T/G polymorphism was conducted by using the report of Schaffer and his colleagues [34]. For molecular analysis of −11391 G/A a PCR-RFLP assay was developed using the following primers:

### Upstream primer

5Á-CATCAGAATGTGTGGCTTGC-3Á.

### Downstream primer

5Á-AGAAGCAGCCTGGAGAACTG-3Á.

The PCR product was cut by MspI restriction enzyme .Enzyme digestion lead to production of 137 and 26 base pairs (with A allele). Digestion products were visualized on Agarose gel (%3.5) which was stained with ethidium bromide Statistical analysis.

### Statistical analysis

In this study, *T*-test, multivariate regression model, POST-Hoc, chi- square and Software version 15 called SPSS were used for data analysis ≤ 0.05 is considered as a data with statistically significance.

## Results

We recorded characteristics of 397 participants of whom 238 subjects suffered from diabetes. Majority of the participants in diabetic group [170 (42.8%)] were females. But in non-diabetic group 36 (9.1%) of participants were female. Mean age of participants was 53 ± 10 and 50 ± 13 years in diabetic and non-diabetic group, respectively. The mean ± SD for WC in diabetics was 91 ± 9 cm, and in non-diabetics was 97 ± 11 cm. All of above analyses had significant p values.

Genotype frequencies of +45 T/G or −11391 G/A for our groups was shown in Table 1. The difference in frequency of adiponectin +45 polymorphism in diabetic and non diabetic cases based on waist circumference was not statistically significant (Table 2). But these results were varied for-11391 polymorphism that was shown in Table 2. After adjusting for presence of diabetes, we observed that this significant difference was independent of sex and age in diabetics (Table 3).

**Table 1:** Genotype frequencies of +45 T/G or −11391 G/A in diabetic and non-diabetic groups

**Variable**	**Diabetics (n = 238)**	**Non-diabetics (n = 159)**	**P value**
**+45 TT**	167 (42.4%)	107 (27.2%)	0.437
**TG + GG**	68 (17.3%)	52 (13.2%)	
**−11391 GG**	221 (67.6%)	83 (25.4%)	0.809
**GA + AA**	16 (4.9%)	7 (2.1%)	

P ≤ 0.05 is considered as significant in *T*-Test.

**Table 2:** The frequency of adiponectin +45 T/G or -11391 G/A genotype with waist circumference in an Iranian diabetic and healthy population

		**Diabetic n (%)**			**Non-diabetic n (%)**		
		**WC ≤ 90**	**WC > 90**	**Total**	**WC > 90**	**WC ≤ 90**	**Total**
	**TT**	75 (32.2)	90 (38.6)	165(70.8)	23 (16.1)	77 (53.8)	100 (69.9)
**+45**	**TG + GG**	31 (13.3)	37 (15.9)	68 (29.2)	16 (11.2)	27 (18.9)	43 (30.1)
	P = 0.985				P = 0.083		
**−11391**	**GG**	95 (40.4)	124 (52.8)	219(93.2)	31(40.8)	40 (52.6)	71(93.4)
	**GA + AA**	12 (5.1)	4(1.7)	16(6. 8)	2(2.6)	3(3.9)	5(6.6)
	P = 0.021*				P = 0.873		

**Legend: WC:** Waist Circumference.

*P ≤ 0.05 in chi-square analysis test is considered as significant.

**Table 3:** Coefficients of multivariate regression model between independent variables and adiponectin +45 T/G or -11391 G/A genotype polymorphisms in diabetics

**Variable**	**WC (>90/≤ 90 cm)**	
	**Odds ratio (CI 95%)**	**P value**
**Sex (M/F)**	1.245	0.459
	(0.697-2.225)	
**Age (>40/≤ 40 yr)**	1.152	0.750
	(0.481-2.760)	
**TG + GG/TT polymorphisms of + 45 T/G genotype**	0.879	0.662
	(0.493-1.567)	
**GA + AA/GG polymorphisms of -11391 G/A genotype**	0.244	0.019*
	(0.075-0.791)	

**Legend: F:** female, **M:** male, **WC:** Waist Circumference, **CI 95%:** Confidence Interval 95%.

*P ≤ 0.05 in multivariate regression model is considered as significant.

## Discussion

We found significantly increased abdominal obesity in GA or AA carriers of adiponectin – 11391 G/A genotype in type 2 diabetes patients. This relation was independent of age and sex. In a study on French population the effects of adiponectin gene polymorphisms at positions (−11377C/G, -11391 G/A, +45 T/G, +276 G/T) were examined with obesity and a strong relationship between adiponectin polymorphisms and severe obesity was observed. Furthermore, they found that haplotypes associated with obesity were related to elevated levels of plasma adiponectin, which indicates a relationship between hyper-adiponectinemia and weight gain [18]. It has also been observed that −11391 G/A polymorphism was associated with higher serum adiponectin levels in obese children [18]. It has been proposed that −11391 A allele might increase the ACDC adipocytes collagen domain-containing activity [18]. In our previous study we have found that the adiponectin −11391 A allele was protective against weight gain only in the women and adiponectin + 45 G allele was protective in both male and female sex [23]. In another study on Danish population the relation between -11391 G/A polymorphism and abdominal obesity has been observed [26]. In one study that performed in a Hispanic population, 18 different adiponectin gene polymorphisms have been investigated with regard to six different criteria for obesity (BMI – Waist circumference - waist to hip ratio - subcutaneous fat tissue-visceral fat-visceral fat to subcutaneous fat ratio) and among them, several polymorphisms were related to obesity located in promoter region [35]. In a non-diabetic Korean population an association between +276 and +45 polymorphisms with serum adiponectin, obesity and insulin resistance has been found [24]. Also, they found a non significant association between +45 polymorphisms and WC. This finding is in line with our finding especially for TT carriers of +45 polymorphism and increased WC. Our results are against the founding’s an study in Italian population which observed a significant relationship between +276 and +45 polymorphisms with insulin resistance and other related indicators such as WC (26). Also, in some other studies, the linear relationship of plasma adiponectin levels with waist circumference is totally obscured [19,36].

Previous studies suggest that the relation between incidence of type 2 diabetes and high blood pressure with adiponectin genes polymorphism is depends on the presence of obesity. It means that these effects are less observed in lean individuals who posses this polymorphism [22]. However, we found higher WC in GA or AA carriers of −11391 G/A polymorphism in diabetes group and also this association was independent of age and sex. Our data is in line with previous reports in French [18], Danish [25] and Hispanic population [35]. However we did not found association with +45 polymorphism and abdominal obesity which confirms the previous report in Korean [29] populations. Therefore, it seems that each polymorphism in adiponectin gene is associated with specific clinical phenotypes. In addition, discrepancies observed in previous studies might be as a result of interaction between different polymorphisms in various conditions.

Finally it seems that more studies in a larger population will be helpful to further confirm our Results. Also study of other common polymorphisms in adiponectin gene in Iranian population is recommended.

## Competing interests

The authors have no competing interests.

## Authors’ contributions

SH-R was the principle investigator, conceived of the study, and wrote draft the manuscript. MMA was the principle investigator, participated in its design and coordination and wrote the draft of the manuscript. OT-M conceived of the study, helped to draft the manuscript and performed the statistical analysis. YR collected the data. JT-B conceived the study. HS collected the data. EA collected the data. All authors read and approved the final manuscript.
